# Challenges of scaling up and of knowledge transfer in an action research project in Burkina Faso to exempt the worst-off from health care user fees

**DOI:** 10.1186/1472-698X-11-S2-S9

**Published:** 2011-11-08

**Authors:** Valéry Ridde, Maurice Yaogo, Yamba Kafando, Kadidiatou Kadio, Moctar Ouedraogo, Marou Sanfo, Norbert Coulibaly, Abel Bicaba, Slim Haddad

**Affiliations:** 1 University of Montreal Hospital Research Centre (CRCHUM), Canada; 2 Department of Social and Preventive Medicine, University of Montreal, Canada; 3 Health Sciences Research Institute [Institut de Recherche en Sciences de la Santé] (IRSS) of the National Centre for Scientific and Technological Research [Centre National de la Recherche Scientifique et Technologique] (CNRST), Burkina Faso; 4 African Agency for Health Education, Research and Expertise [Agence de Formation, de Recherche et d'Expertise en Santé pour l'Afrique] (AFRICSanté), Burkina Faso; 5 Society for Public Health Study and Research [Société d’Études et de Recherche en Santé Publique] (SERSAP), Burkina Faso; 6 Burkina Faso Ministry of Health [Ministère de la santé], Burkina Faso

## Abstract

**Background:**

Systems to exempt the indigent from user fees have been put in place to prevent the worst-off from being excluded from health care services for lack of funds. Yet the implementation of these mechanisms is as rare as the operational research on this topic. This article analyzes an action research project aimed at finding an appropriate solution to make health care accessible to the indigent in a rural district of Burkina Faso.

**Research:**

This action research project was initiated in 2007 to study the feasibility and effectiveness of a community-based, participative and financially sustainable process for exempting the indigent from user fees. A interdisciplinary team of researchers from Burkina Faso and Canada was mobilized to document this action research project.

**Results and knowledge sharing:**

The action process was very well received. Indigent selection was effective and strengthened local solidarity, but coverage was reduced by the lack of local financial resources. Furthermore, the indigent have many other needs that cannot be addressed by exemption from user fees. Several knowledge transfer strategies were implemented to share research findings with residents and with local and national decision-makers.

**Partnership achievements and difficulties:**

Using a mixed and interdisciplinary research approach was critical to grasping the complexity of this community-based process. The adoption of the process and the partnership with local decision-makers were very effective. Therefore, at the instigation of an NGO, four other districts in Burkina Faso and Niger reproduced this experiment. However, national decision-makers showed no interest in this action and still seem unconcerned about finding solutions that promote access to health care for the indigent.

**Lessons learned:**

The lessons learned with regard to knowledge transfer and partnerships between researchers and associated decision-makers are: i) involve potential users of the research results from the research planning stage; ii) establish an ongoing partnership between researchers and users; iii) ensure that users can participate in certain research activities; iv) use a variety of strategies to disseminate results; and v) involve users in dissemination activities.

## Introduction

Access to health care is restricted particularly by geographic, sociocultural and financial factors [1]. In Africa in general, and in Burkina Faso specifically, patients must pay for health care services. There is no national health insurance. In a country in which 40% of the population lives below the poverty line, such health care user fees cause major inequalities. For example, only 39% of poorest women give birth attended by qualified personnel, compared to 91% of well-off women [2]. Eighty percent of poor people are forced to go into debt or sell their assets to pay for health care [3].

In light of this, health policies in Africa have always advocated exempting the indigent, who are consistently unable to pay for care [4], from point-of-service user fees [5]. However, very few countries have been able to implement these types of systems [6]. We have little knowledge in the African context, where much of the economy is informal, of the criteria used to determine indigence and of the processes that can help identify the indigent. However, decision-makers in Burkina Faso have been calling for the knowledge needed to organize such measures for the past 20 years [7].

## The research project

To satisfy this need for knowledge, an action research project was initiated in 2005. Our previous research showed that: i) villagers’ knowledge about each other can be used to identify the indigent; ii) health centre management committees (COGESs), which manage the money paid by users, have a certain financial capacity to exempt the indigent from user fees; and iii) trials need to be conducted in the national context to generate local knowledge with significant potential to influence national decision-makers [8,9].

This action research project [10] was launched in 2007 in the Ouargaye health district (260,000 inhabitants, Centre-East region of the country). The objective was to study the feasibility and effectiveness of a community-based, participative process for exempting the indigent from health care user fees that would be based on endogenous and sustainable funding. Endogenous funding means that, in accordance with the intent of the Bamako Initiative and Burkina Faso's statutory instruments, user fees exemptions for the indigent are funded by profits from consultation fees and medication sales collected from patients using health centres who are able to pay.

Between 2005 and 2007, a participative process was carried out to plan the outlines of the action project and the study. All central and peripheral actors from the Ministry of Health and the Ministry of Social Action, as well as community representatives and other local leaders, were invited to take part in this planning process.

The action project, carried out in partnership with the team from the health district and the provincial social action department, consisted in asking villagers in half of the district's health centres (n = 10) to identify the people whom they considered to be indigent [11]. In 2007, village selection committees (VSC) were created in each village (n = 124), made up of seven people who were mandated to draw up a list of indigent people. Nearly 600 people were listed. Because the 10 COGESs would be funding this exemption from their annual profits to ensure sustainable support, they were asked to validate the list. They retained 46% of the people on the list (n = 269), or just under 3 inhabitants per 1,000. Approximately half of these people were women and more than 60% were over the age of 50. All these people received an official card from the provincial social action department exempting them from paying for primary care and care provided at the district hospital. Among them, 40% used health services in the following year, at an average cost of 1,300 F CFA (2.60 USD) per consultation.

Because health care workers found it difficult to identify the indigent based on a list of 20 criteria that we provided, those working in other health centres in the district that were not involved in the trial asked that it be reproduced. Therefore, in 2010, the trial was expanded to include all villages (n = 257) and health centres (n = 26) in the district. The village selection committees then identified 2,650 indigent people, of which the COGESs retained 1,100, or 41%. Also, following a study trip to Ouargaye in 2008, teams from two other districts in the country (Dori and Sebba) and from two districts in neighbouring Niger (Mayahi and Tera) tested the action project in 2009. In early 2011, it was extended to all the villages in Dori and Sebba. The village selection committees identified 1,400 and 865 indigents, respectively, of whom the COGESs retained 292 and 312. In Niger, the pilot project in nine health centres resulted in indigence cards being distributed to 244 people.

A number of research activities were carried out in conjunction with this action project. While space constraints do not allow us to describe in detail here all the methods used, the fundamental point to note is that we employed both qualitative and quantitative methods and assessed not only the appropriateness of the action project, but also its processes and effectiveness. A multidisciplinary team from Canada and Burkina Faso was set up to answer three main questions: i) Was the action project adapted to the social context, well received by stakeholders, and non-stigmatizing of the indigent? ii) What process was used to implement the action, and what were its strengths, weaknesses and local adaptations? and iii) Did the action project make it possible to reduce both inclusion (selecting non-indigent people) and exclusion (leaving out indigent people) biases?

## Results and knowledge sharing

### A suitable, consensual and participative process

The research results showed that the community-based selection process was perfectly adapted to the social context and well received by residents and health care workers. Thus, we showed that communities are able to identify the indigent in their villages. In rural areas, therefore, it is not necessarily useful to wait for a list of indigence criteria to be defined. However, the request from nurses, who have been asking for these criteria for the past 20 years, to scale up this pre-identification method to the entire district is additional proof of the relevance of the community-based approach.

### Needs beyond access to health care

While indigence status did not lead to social stigmatization, the process revealed that access to health care was just one of many needs (not always perceived), even though the action research focused solely on that aspect. Indeed, the research action project focused on financial access to health care as a gateway to understanding why health services are used very little, if at all, by the indigent. It responded to a need that had long been widely expressed by those working in Burkina Faso’s health system and by residents, but never studied [9]. In fact, to our knowledge, this was the first time this type of community-based process had been tested and that indigence cards were distributed in rural areas. Therefore, it introduced a major innovation. However, researchers and stakeholders also quickly discovered the limitations of an action project centred on the financial determinants of access to care. By adapting a longitudinal case study method that is both qualitative and quantitative [12], we showed that the living conditions of a sample of 20 indigent people monitored for 12 months after receiving the card had fundamentally not changed. Also, their use of health centres was constrained in particular by very precarious health status, social isolation, lack of transportation and low priority given to health compared to their need to eat. Speaking about an indigent person, one interviewer observed, "*He doesn't care about health care; he's thinking about where he'll get his next meal and where he'll sleep*."

The scientific literature has long shown that geographic or social distance between patients and health care workers also affects use of services (not to mention, of course, the social determinants of health). The researchers were clearly aware of these facts but could not attempt to resolve all access issues in a single action research project because of the difficulty in finding ways to conduct research in this area and because of the chosen approach. However, the research in Ouargaye helped bring to light once again the two limitations of actions centred solely on financial access to health care: i) the use of services is constrained by aspects other than just payment for health care, and ii) the health of indigent people is affected by multiple factors outside of the health care system. While this is by no means a new scientific discovery, these results argue for the organization of more holistic and interdisciplinary action research projects to improve the living conditions of the worst-off. However, there again, we come up against the constraints of health research funding methods, which hardly favour this type of approach.

### Stronger but limited local solidarity

The fact that the principle of solidarity was brought to light again through this endogenous funding was welcomed by all stakeholders. However, this local solidarity obviously has limits. Remember that in Burkina Faso, nearly half of the population (46%) lives on less than 280 F CFA (0.50 USD) per day. The communities made very few inclusion errors because they selected people who were truly very poor. On the other hand, in Ouargaye, they selected only a tiny portion of the poor population (0.36%) and the extremely poor population (0.78%), whereas the proportion of people in the country living below the extreme poverty line is 9% [13]. The results were approximately the same in Dori and Sebba, where the social context is very different but indigent coverage remained very low as well: 0.13% and 0.36% of the general population, respectively. The main explanation for this restricted selection is that the exemption is covered entirely by local funding. Taking into account their revenue-generating capacity, while the communities could have selected a few more indigents, it would not have been right to ask poor people to fund health care access for all the very poor people. The health centres’ annual profits could cover the needs of 1,800 indigents per year, which is not negligible, but would represent only 0.7% of the population of the Ouargaye district. Current efforts are already outstanding and show that local solidarity can be mobilized. However, the government, indisputably the supreme power, must guarantee health care access to indigent people. This action research project contributes to the development of knowledge on this issue in Africa [14,15] and has shown that there is now a feasible, suitable and effective process for selecting indigent people. The government and its financial partners must now mobilize more resources to increase the coverage of support for indigent people. At a minimum, the 9% of the population considered to be extremely poor should be covered, and communities could be mobilized to identify these people. Moreover, an attempt should be made to implement a national equity fund financed by the government, its partners and local communities to support a greater number of indigent people.

### A variety of knowledge transfer strategies

**Figure 1:**
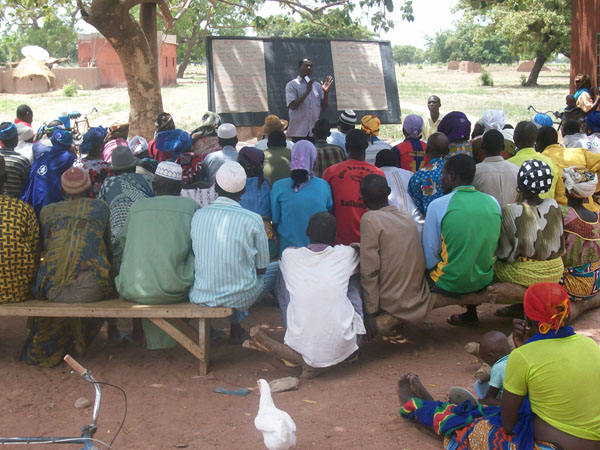
**Community forum for presentation of results**. Community forum for presentation of results conducted by a member of the Ouargaye health district team in the village of Yourga (June 2009)

To ensure the results would be both useful and used, we developed multiple knowledge transfer strategies:

i) *Very early involvement of potential users of the results in defining the action project and the outlines of the study*: Beginning in the planning stage, all the main potential stakeholders in the action research project were involved (regional department of health, district management team, the Burkinabé Public Health Association, a key representative from the national health development program (PNDS), and health centre management committees).

ii) *Involvement of local partners of the action research project in the processes of research and results presentation*: Some members of the district management team and managers in the social action department were involved in many activities (choosing pilot project sites, informing and training community stakeholders in all the health centres, making and signing indigence cards, etc.).

iii) *Presentation of results to communities and at national meetings of the Ministry of Health and stakeholders*: It was not enough to present the results in scientific arenas; the research results were also shared at community forums (Figure 1), at district team meetings, and at meetings with all health care workers in the district, at the biannual conference of all of the country’s regional health directors and district chief medical officers, and at the national round table on social protection [Table ronde nationale sur la protection sociale] (http://sites.google.com/site/protectionsocialeauburkinafaso/), among others.

iv) *Presentation of results at national*, *regional and international scientific conferences*: To increase the number of dissemination processes, scientific presentations were given in Burkina Faso, Senegal, France, Canada, Switzerland, Belgium, etc. A workshop was specifically organized at the First Global Symposium on Health Systems Research held in Montreux in November 2010.

v) *Organization of an international workshop in Burkina Faso*: A workshop was organized in October 2010 to encourage stakeholders in the field to share their experiences and to increase the likelihood of getting decision-makers to integrate this issue into public policy. More than 60 people participated in the two-day workshop, and presentations were made on similar experiences in Burkina Faso, Cambodia, Ivory Coast, Ghana, Mali, Mauritania and Niger.

vi) *Preparation and distribution of a policy brief*: We know decision-makers do not always have time to read long reports or scientific articles. Therefore, we also prepared a short 1,500-word text (four pages with illustrations) summarizing the main results of this action research project. The document was translated into English and widely distributed, both as a hard copy version in Burkina Faso (In French) and as an electronic version across the networks interested in this topic.

vii) *Production of a 26-minute documentary*: We produced a documentary film to raise awareness of the issue of indigence and to have a visual tool that would attract attention. A film director from Niger, who had also produced a documentary on the issue of free health care for another one of our research projects, came to Ouargaye to give all the stakeholders involved an opportunity to express themselves. The film will be broadcast within the country and may also be used by the director in international competitions.

All of these knowledge transfer products are available online at: http://www.medsp.umontreal.ca/vesa-tc/indigents.htm.

## Partnership achievements and difficulties

### Interdisciplinary joint research project

The challenges involved in the partnership between researchers were more of an interdisciplinary nature than about North-South differences. The lessons learned were mutual, and the focus was on the complementarity of theoretical and methodological approaches. For example, anthropologists most often use a very inductive process in conducting their research, whereas researchers in evaluation and public health generally organize their data using an analytical framework. In this case, the processes were evaluated based on the intervention theory (elements describing the logic of the action) without necessarily restricting the collection of qualitative data to this aspect alone. Using mixed methods to evaluate the action and to understand the process was very useful [16]. The study of 20 cases of indigence, as described above and adapted from a methodological approach applied elsewhere [12], enabled us not only to better understand their life trajectories, but also to calculate their spending levels. The qualitative data was also useful to counterbalance the necessarily reductionist view of poverty that is required to evaluate the effectiveness of the targeting [13], from an economic standpoint [17]. Calculating the health centres’ accounting data [18] was also useful to inform the COGESs of their financial capacities and to help them understand the extent to which they could support the indigent.

More relevant to this text is a discussion of the partnership between the researchers, decision-makers and communities, in which lies the originality of the action research project.

### Close relationships between researchers and local users

The researchers involved in this project and concerned about how the knowledge would be used [19] saw the participation of central and peripheral representatives of the Ministry of Health and the Ministry of Social Action from the outset, when the action was first being considered and then being planned, as a condition that would promote knowledge utilization. Community involvement was similarly viewed, but it was also, and primarily, aimed at ensuring the action would be adopted and sustained. Although this latter aim was relatively successful, it should be noted that partnership with these representatives was often arduous, particularly at the central level.

Clearly, the closeness of the relationships between researchers and local decision-makers or practitioners was a positive factor for the application of knowledge [20]. Their interest was increased and the partnership strengthened by the fact that a concrete solution was being proposed to problems they encountered on a daily basis [21]. The participative process, the regular sharing of the research results and the continuous presence of the research coordinator in this isolated rural area for the first two years of the project are also factors that help to explain this success. Likewise, regular and sustained contact was essential with members of an NGO operating in the four districts (in Burkina Faso and Niger) that had decided to reproduce the experience.

### National decision-makers not very concerned about support for the indigent

On the other hand, the adoption of the strategy by central decision-makers, those who write policies, was more problematic despite several attempts and presentations of the study’s evidentiary data. Like the inter-ministerial committee on support for the indigent created in 2005 that rarely manages to meet, we ourselves, after two years of trying to organize a national forum on the subject, were unable to involve the central decision-makers. They never wanted to assume the leadership on this issue. We eventually decided to take it upon ourselves by inviting them (with little success) to the abovementioned international workshop (see v) without involving them in its organization. The observation made several years ago regarding the decision-makers’ lack of interest in this issue appears to still apply [9,22]. A recent evaluation of the National Health Development Program [Programme National de Développement Sanitaire] (2001-2010) came to the same conclusion: “Certain studies on indigent people in districts are not promoted at the national level” [23]. Central decision-makers do not receive requests from indigent people on a daily basis and are preoccupied with numerous meetings, workshops and other assignments. At a March 2010 meeting organized by a group of journalists concerned about the welfare of the worst-off, the Ministry representatives did not mention the Ouargaye experience, whereas they had been informed of it at the previous Ministry meeting on health in late 2009 (CASEM), as had been all of the central directors. However, in its analysis of the national health situation in 2010, prior to the reformulation of its national health policy, the Ministry recognized that “research initiatives on this subject were developed, particularly in the Sahel and Centre-East regions” [24]. However, the research we conducted in the most recent years of our involvement in this issue shows that there is no true political will to resolve this complex problem, either nationally or internationally. For example, at the national level, the country has adopted an ambitious policy to fund deliveries within the national budget, according to which 20% of women considered indigent would be exempted from user fees for this service. However, health care workers and managers are either not aware of this provision, or are waiting for the government to provide them with criteria. Yet the implementation document for this policy stipulates that one of the exemption criteria is “indigent status recognized by legislation or by the community” [25]. Ministry of Health representatives often bounce the issue back to their colleagues at the Ministry of Social Action, calling on them to propose solutions. Although this Ministry is the only one authorized to issue indigence certificates, it has few resources to carry out this mandate; it has no agents outside the cities and, despite the fact that the agents are the experts on indigence, they have no shared vision of the issue and no standard selection criteria [26]. So, all that would be needed is a decision to extend the Ouargaye experience.

## Lessons learned

With regard to the knowledge updated by this study about support for indigent people, our experience has shown that:

• Communities are able to select indigent people;

• The community-based, participative process adopted did not lead to social stigmatization of the indigent;

• Lowering the financial barrier to health care access is not enough in itself to satisfy the many needs of the indigent;

• Conflicts of interest between providing free health care and having inadequate revenue-generating capacity reduce the coverage potential of the community-based approach for indigent selection;

• National decision-makers’ have no interest in supporting indigent people.

With regard to the application of knowledge and the partnership between researchers and peripheral decision-makers, we have learned the importance of:

• Involving potential users of the results from the start, when planning the organization of the research project;

• Ensuring a continuous and sustained partnership between researchers and users;

• Making sure users are able to participate in some research activities;

• Using a variety of strategies to disseminate the research results;

• Involving users in the dissemination activities.

## Competing interests

The authors declare that they have no competing interests.

## Author contributions

VR, SH, MY and AB wrote the research protocols. YK and KK coordinated the action project with MS and NC. MY organized the collection of qualitative data with VR, YK, KK and AB. MO organized the collection of quantitative data with AB, VR and YK, and the initial analyses with SH and VR. The quantitative analyses were performed by SH and VR with the help of Béatrice Nikiema. The qualitative analyses were conducted by MY and VR. VR wrote the draft versions of the article. All of the authors read, revised and approved the final article.
